# Vitexin Possesses Anticonvulsant and Anxiolytic-Like Effects in Murine Animal Models

**DOI:** 10.3389/fphar.2020.01181

**Published:** 2020-08-11

**Authors:** Denise Dias de Oliveira, Cassio Prinholato da Silva, Bruno Benincasa Iglesias, Renê O. Beleboni

**Affiliations:** ^1^Department of Biotechnology, University of Ribeirão Preto, Ribeirão Preto, Brazil; ^2^School of Medicine, University of Ribeirão Preto, Ribeirão Preto, Brazil

**Keywords:** epilepsy, anxiety, etnopharmacology, flavonoid, γ - aminobutyric acid (GABA), glutamate

## Abstract

Different types of epilepsy and forms of pathological anxiety have been described as significant neurological disorders that may exist as comorbidities. Some of those disorders share the association of affected limbic areas/neuropathological triggers as well as the use of drugs for their clinical management. The aim of this work was to investigate the anticonvulsant and anxiolytic properties of the vitexin (apigenin-8-C-glucoside), since this compound is a flavonoid usually found as one of the major constituents in several medicinal plants claimed as anxiolytics and/or anticonvulsants. This investigation was performed by the use of a series of classical murine animal models of chemically induced-seizures and of anxiety-related tests (open-field, elevated plus-maze, and light-dark box tests). Here, we show that the systemic administration of vitexin (1.25; 2.5 and 5 mg/kg; i.p.) exhibited selective protection against chemically-induced seizures. Vitexin did not block seizures evoked by glutamate receptors agonists (NMDA and kainic acid), and it did not interfere with the latencies for these seizures. Conversely, the same treatments protected the animals in a dose-dependent manner against the seizures evoked by the Gabaergic antagonists picrotoxin and PTZ and rise the latency time for the first seizure on non-protected animals. The higher dose of vitexin protected 100% of animals against the tonic-clonic seizures triggered by GABA antagonists. The results from open-field, elevated plus-maze, and light-dark box tests indicated the anxiolytic properties of vitexin at similar range of doses described for the anticonvulsant action screening. Furthermore, these results pointed that vitexin did not cause sedation or locomotor impairment on animals. The selective action of vitexin against picrotoxin and PTZ may reinforce the hypothesis by which this compound acts mainly by the modulation of GABAergic neurotransmission and/or related pathways. This could be useful to explain the dual activity of vitexin as anticonvulsant and anxiolytic, and highlight the pharmacological interest on this promising flavonoid.

## Introduction

Epilepsy is a progressive chronic disorder characterized by an abnormal and synchronous neuronal firing associated with recurrent and unpredictable seizures (England et al., 2012). It affects more than 50 million people worldwide. The current antiepileptic drug arsenal fails to evoke a positive response in about 30% of diagnosed cases of epilepsy (World Health Organization, 2019a). Patients suffering from different types of epilepsy are often affected by psychiatric and behavioral comorbidities such as mood disorders, anxiety, psychoses, motor disorders, cognitive deficits, and social dysfunctions (Salpekar and Mula, 2018). Besides the broad epidemiological relevance of anxiety disorders at different segments of our modern society, anxiety disorders are usually observed in a large number of epileptic patients, which represents additional negative impacts in their already compromised quality of lives (Gandy et al., 2015). It is well known that the alleviation of anxiety symptoms may have a positive effect in the progress of treatment of epileptic patients contributing to ameliorate their general health condition (Fisher and Noble, 2017).

The pathophysiological mechanism related to the onset and maintenance of different types of epilepsy/seizures, includes the imbalance between excitatory (glutamate) and inhibitory (γ-aminobutyric acid (GABA) synaptic activities that are located at different brain areas (Bialer and White, 2010). Some brain networks are commonly involved at prevention or appearance of seizures as well as in the regulation of behavior and mood (Kwon and Park, 2014). Although the huge differences in the molecular and cellular bases involved in onset and development of epilepsy and anxiety, the same imbalance between GABA and Glutamate is also related to anxiety (Masneuf et al., 2014; Gauthier and Nuss, 2015). Of course, several intervenient factors can differentially contribute to the neuropathology underlying both disorders, particularly in terms of magnitude, recruited structures, and brain areas (Martin et al., 2009; Wu et al., 2018). In any case, the screening for new antiepileptic/anxiolytic drugs that are able to modulate the inhibitory and/or excitatory neurotransmission pathways may be worth for the treatment of epilepsy and/or anxiety (Moto et al., 2018).

Vitexin (apigenin-8-C-glucoside) has received great attention by presenting a wide range of pharmacological effects. Vitexin has been found, in some cases as the major constituent, at different medicinal plants potentially useful to treat anxiety and/or epilepsy (Nassiri-Asl et al., 2007; He et al., 2016). More specifically, recent studies have shown the protective effects of vitexin against some neurological and psychiatric diseases (Abbasi et al., 2013; Can et al., 2013; Guimarães et al., 2015). In spite of the interesting pharmacological properties of vitexin; only a few and preliminary efforts have been conducted to prove its potential anxiolytic and anticonvulsant activities. Therefore, the main aim of this work is to investigate these potential effects using a complementary set of experiments at a well-established animal model arrangement.

## Materials and Methods

### Vitexin and Other Drugs

Vitexin (apigenin-8-C-glucoside) was purchased from Cayman Chemical Company, USA (CAS Number 3681-93-4, purity degree ≥ 98%). The proconvulsant drugs N-Methyl-D-aspartate (NMDA) (CAS Number: 6384-92-5; ≥ 98%); kainic acid (CAS Number: 58002-62-3; ≥ 98%); Pentylenetetrazol (PTZ) (CAS Number: 54-95-5) and picrotoxin (CAS Number 204-716-6) were purchased from Sigma Aldrich, St. Louis, MO. Diazepam (DZP) (injectable solution in 0.9% saline) and dimethyl sulfoxyde (DMSO) were acquired from União Química (Brazil) and Synth (Brazil), respectively.

### Animals and Experimental Conditions

All experiments were carried out using male adult Wistar rats within 4 to 5 weeks of age weighing about 150 to 180 g. Rats were housed five per cage in a controlled condition of humidity and temperature under 12 h light/dark schedule at 6 am/6 pm. They were allowed to acclimatize to our host facilities for at least 3 days prior to any experimental manipulation. Chow and water were provided *ad libitum*. The procedures were in accordance with the University of Ribeirão Preto Ethic Committee (approval number 11/2015) and with the Guide for the Care and Use of Laboratory Animals (National Research Council, US).

The animals were divided in control and experimental groups (*n*=06) in all sets of experiments. All the experiments were carried out in an acoustic isolated room between 1:00 to 5:00 p.m. All apparatus or arenas used in experiments were cleaned with 70% ethanol after observation of each animal in all of the procedures. Rats were habituated to the testing room for 30-min before experimental procedures. All experiments were recorded by a digital video camera and copies of files kept in our laboratory as official documents for public consultation.

## Assessment of Anxiolytic-Like Effects of Vitexin

The anxiolytic activity of vitexin was evaluated by elevated plus-maze, light-dark box, and open-field tests. The behavior of animals on each experimental apparatus for anxiolytic effects was observed 30 min later after control or experimental treatments. For all experiments, Diazepam 2 mg/kg (dissolved in 0.9% saline) (i.p.) served as positive control while 1% DMSO in 0.9% saline (i.p.) and 0.9% saline solution (i.p.) served as controls. Experimental groups were composed of animals treated with different i.p. doses of vitexin (0.75, 1.25 and 2.5 mg/kg). Doses of vitexin were selected based on pilot scale tests while the dose of DZP were based on previous scientific reports (Kazdoba et al., 2015; Zemdegs et al., 2018)

### Elevated Plus-Maze Test

The elevated plus maze (EPM) was conducted in accordance with the method validated by Pellow et al. (1985).The maze comprised of two wood open arms (50 × 10 cm) surrounded by a short (0.5 cm) acrylic edge to avoid falls and two wood enclosed arms (50 × 10 × 40 cm) arranged such that the two open arms were opposite to each other. The arms were connected by a central platform (10 × 10 cm) and the maze was kept 50 cm above the floor. Each rat was placed in the middle compartment (head facing an open arm) and allowed to freely explore the apparatus for 5 min. It was measured (i) the time spent(s) in the open and closed arms of the EPM, (ii) the percentage of time spent in open arms, (iii) protected and unprotected stretch attend postures, and (iv) protected and unprotected head dipping events. Stretch-attend was defined when the animal stretches forward and retracts without moving its feet. Head dipping was defined as the exploratory movement in which the rodent head protruding over the edge toward the floor on the open arms. Behaviors were defined unprotected when they were exhibited in the open arm region of the EPM and were protected if it occurs in closed arms or central platform of the maze (Walf and Frye, 2007).

### Open Field Test

The open field test was performed according to the method previously described (Funchal and Dani, 2014).The open-field used was a circular arena measuring 60 cm in diameter with 50-cm high circular acrylic wall (OP0199, Insight). The apparatus floor was divided into 12 squares (4 squares corresponding a central zone and 8 squares to the peripheral zones).Every animal was placed in the central region of the open field, and the number of lines crossed (all four limbs), rearing, grooming, and the times spent(s) in the central zone of the apparatus were recorded for 5 minutes. Also, for an indirect measurement of locomotor performance of animals, the number of crossings was checked in time blocks along the experiment comparing control and experimental groups, since the animals usually explore more of the apparatus in the first blocks of time and less at the end of the task, as they habituate.

### Light-Dark Box Test

The light-dark box consist of a *box* (46 x 27 x 30 cm) divided into a dark and illuminated compartment (EP 158, Insight, fluorescent lamp- 20 W). The chambers were connected by a small opening (7.5 × 7.5 cm) in the middle of the wall separating the two chambers. Rats were placed in the middle of the light chamber and then released to explore for 5 min (Hascoet and Bourin, 1998).The time that rats spent in the light/dark compartments and the number of transitions between them were recorded. Transition was defined as the placement of all four paws in the entry chamber.

## Assessment of Anticonvulsant Activity of Vitexin

The anticonvulsant effect of vitexin was evaluated against chemically-induced seizures by *intraperitoneal (i.p.) injection* of proconvulsant agents: NMDA, 150 mg/kg; kainic acid, 30 mg/kg; picrotoxin, 6 mg/kg; and PTZ 90, mg/kg. NMDA, kainic acid, picrotoxin, and PTZ were dissolved in 0.9% saline while vitexin dissolved in 0.9% saline containing dimethyl sulfoxyde – DMSO 1%. The animals control and experimental groups were divided according to the assessment of anxiolytic-like effects of vitexin, except by the range of vitexin i.p. doses, which were slightly up changed for anticonvulsant screening (1.25, 2.5, and 5 mg/kg). Doses of vitexin were selected based on pilot scale tests while the dose of DZP were selected based on previous scientific reports, as considered above for anxiolytic-related tests. Thirty minutes after the administration of each proconvulsant drug, each animal was maintained individually in a transparent *acrylic arena* (60 cm × 40 cm). The rats were observed for 30, 40, 40, and 120 min respectively for kainic acid, NMDA, picrotoxin, and PTZ assays. It was evaluated the latency time to the first generalized tonic-clonic seizure and the incidence/number of animals exhibiting generalized tonic-clonic seizures (Funchal and Dani, 2014). Animal behavior seizure rating was classified/certified according to the Racine’s scale, modified by Pinel and Rovner (Pinel and Rovner, 1978). Generalized tonic-clonic seizures were considered as those rating score 7 or more in accordance to this scale and analyses of our video-recorded files.

### Statistical Analysis

Except by the Open-Field test which was performed one and two-way ANOVA test, other experiments statistical analyses were performed using One-way ANOVA, followed by the Tukey post-hoc test (GraphPad Prism; version 7.0, Graphpad Software, USA). Data were expressed as the means ± standard error of mean (SEM) (*n*=06/group). A level of p< 0.05 was accepted as statistically significant.

## Results

### Anxiolytic-Like Effects of Vitexin

#### Elevated Plus-Maze

**Figure 1:**
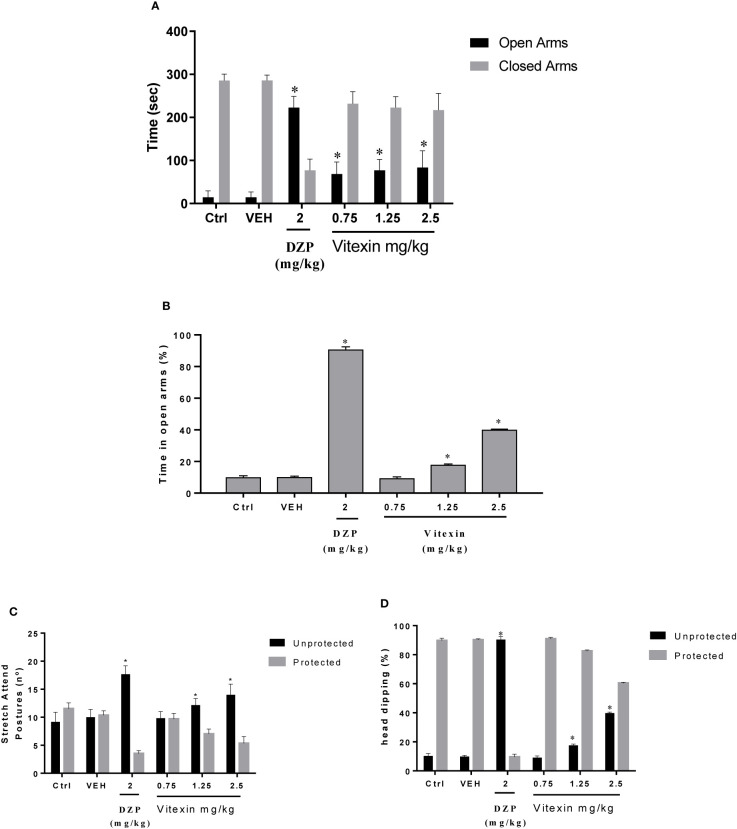
Elevated Plus Maze. **(A)** Total time spent on the open and closed arms, **(B)** percentage of time spent in opens arms, **(C)** protected and unprotected stretch attend postures and **(D)** protected and unprotected head dipping between the groups treated with different doses of vitexin, diazepam (DZP), and vehicle (VEH), compared with control group (Ctrl - Saline Control). Significantly different from saline control group: ^∗^*p* < 0.05. One-way ANOVA; Tukey *post hoc* test.

The amount of time (sec) spent in the open and closed arms and percentage of time spent in open arms in the elevated plus maze are shown in **Figure 1**. The *post hoc* test showed that the time spent on the open arms was longer (*p* < 0.05) in the diazepam treated group (positive control). Also, the data shows that all doses of vitexin increased significantly the time spent in the open arms compared to the saline control (F_(5,60)_ = 103.9; p<0.05). Pretreatment with vitexin produced a significant increase in unprotected head dipping (F_(5,30)_ = 74.25; p<0.05) and unprotected stretch-attend postures (F_(5,30)_ = 27.99; p<0.05).

#### Open Field Test

**Figure 2:**
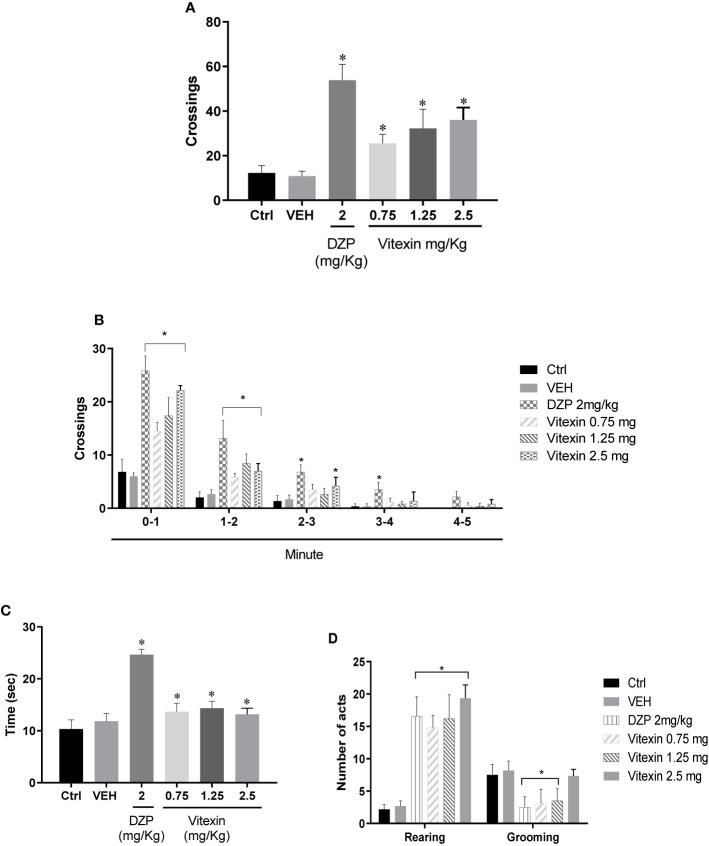
Open Field Test. **(A)** number of crossings made in the open field, **(B)** Crossings in function of the time, **(C)** time spent in the center and **(D)** rearing and grooming behavior of the open field by different doses of vitexin, diazepam (DZP), and vehicle (VEH), compared with control group (Ctrl—saline control). Significantly different from saline control: ^∗^*p* < 0.05. For data in **(A, C** and **D)** was performed one-way ANOVA and in **(B)** was performed two-way ANOVA. For all data were performed Tukey *post hoc* test.

The number of line crossings during open field test showed that vitexin increased locomotion on apparatus (F_(5,26)_ = 45.33; p<0.05) (**Figure 2A**). Vitexin significantly improved locomotion mainly in the first 3 minutes of experimentation when compared with the control group (F _(20, 140)_ = 26,23 p<0.05) (**Figure 2B**). In the open field test, animals treated with vitexin (all doses) spent more time on the center of arena when compared with those from the control group (F_(5,30)_ = 76.82; p<0.05) (**Figure 2C**). Vertical activity behaviors (rearing and grooming) are shown in **Figure 2D**. The analysis of total number of the rearing showed significantly higher for vitexin treatments than that in the control group (saline) (F_(5,27)_ = 67.49; p<0.05). The *post hoc* test revealed that vitexin 0.75 and 1.25 mg/kg reduced grooming behaviors (compared with the control group) in a way similar to the diazepam (F_(5,27)_ = 40.02; p<0.05) (**Figure 2D**).

#### Light-Dark Test

**Figure 3:**
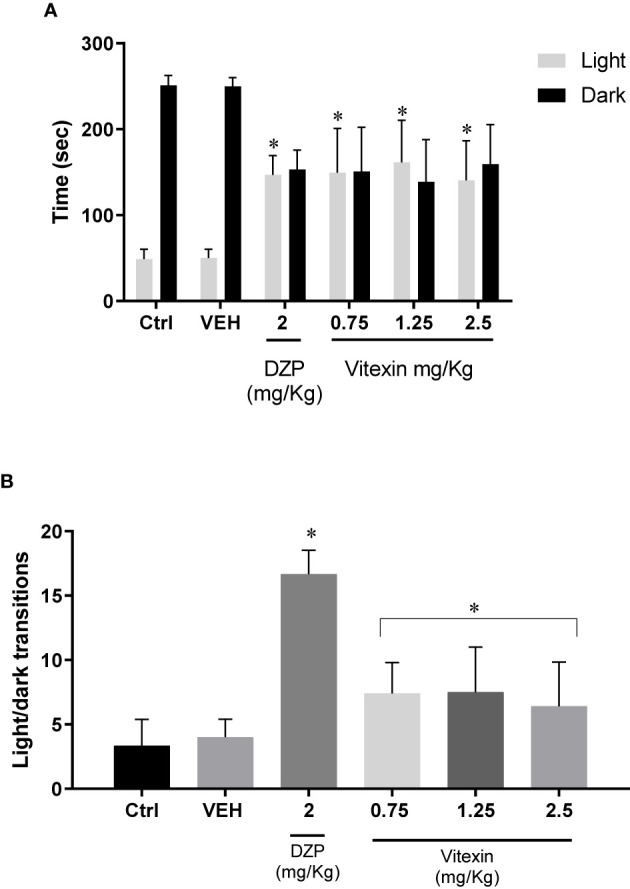
Light dark box test. **(A)** times spent(s) on light/dark area and **(B)** light/dark transitions by different doses of vitexin, diazepam (DZP) and vehicle (VEH) compared with control group (Ctrl—saline control). Significantly different from saline control: ^∗^*p* < 0.05. One-way ANOVA; Tukey *post hoc* test.

Vitexin at the dose of 0.75, 1.25, and 2.5 mg/kg and diazepam (2 mg/kg) induced a significant increase in the time spent by rats on the illuminated side of the apparatus compared with the saline control group (F_(5;50)_ = 26.87; p<0.05) (**Figure 3A**). Analysis of the light/dark transitions revealed significant greater number of transitions for animals treated with vitexin than in the control group (F_(5,28)_ = 21.33; p<0.05) (**Figure 3B**).

### Anticonvulsant Screening

**Figure 4:**
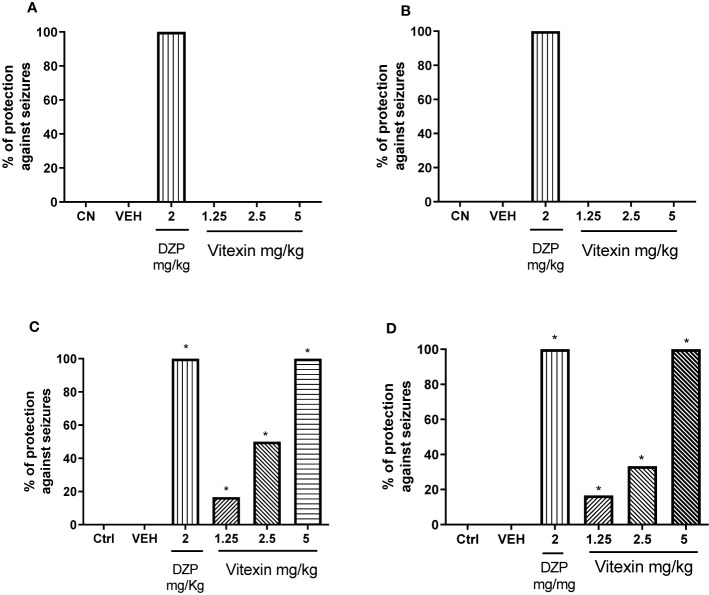
Percentages of animals protected against seizures induced by **(A)** kainic acid 150 mg/kg, **(B)** NMDA 150 mg/kg, **(C)** picrotoxin 6 mg/kg and **(D)** PTZ 90 mg/kg in function of different doses of vitexin, diazepam (DZP), and vehicle (VEH) compared with control group (Ctrl-Saline Control). Significantly different from control: *p < 0.05 versus saline control group. One-way ANOVA; Tukey *post hoc* test.

Acute administration of vitexin prior to kainic acid and NMDA injections did not protect animals against seizures. However, clonic seizures induced by i.p. administrations of PTZ and picrotoxin were inhibited by all doses of vitexin (**Figures 4A, B**). Vitexin significantly protected animals against seizures and showed 16.66, 50 and 100% of protection against picrotoxin-induced seizures (**Figure 4C**) and 16.66%, 33.33%, and 100% against PTZ-induced seizures (**Figure 4D**), respectively, on the doses of 1.25; 2.5, and 5 mg/kg. Therefore, the anticonvulsant activity of vitexin exhibited a dose-dependent profile on PTZ and picrotoxin assays. In addition, the latency to to the first seizure induced by PTZ and picrotoxin was significantly higher for groups treated with vitexin, particularly on the dose of 2.5 mg/kg (**Table 1**).

**Table 1:** Latency to onset of seizure (score 7-8) on vitexin treatments.

Convulsant Agent	Latency* *to* *onset of seizure (seconds)					
	Ctrl	VEH	DZP (2 mg/kg)	Vitexin (1.25 mg/kg)	Vitexin (2.5 mg/kg)	Vitexin (5 mg/kg)
NMDA (150 mg/kg)	4409.50 ± 92.48	4326.30 ± 43.49	NS	4403.83 ± 98.63	4514.50 ± 63.59	4548.50 ± 70.52
Kainic acid (30 mg/kg)	4491.33 ± 77.88	4546.33 ± 77.52	NS	4693.0 ± 74.59	4404.83 ± 116.83	4468.72 ± 124.72
PTZ (90 mg/kg)	77.4 ± 14.63	78.00 ± 7.38	NS	53.66 ± 9.07^#^	141.2 ± 29.08*^#^	*^#^ NS
Picrotoxin (6 mg/kg)	1101.0 ± 47.29	1103.66 ± 47.99	NS	745.8 ± 65.35*^#^	1396.6 ± 43.14*^#^	*^#^ NS

∗p < 0.05 versus control group (Ctrl – Saline Control) and ^#^p < 0.05 between different doses of vitexin. One-way ANOVA; Tukey post hoc test. NS, no seizure.

## Discussion

In the present study we demonstrated that vitexin presents both anxiolytic and anticonvulsant properties which are possibly mediated by modulation of GABAergic neurotransmission and/or related synaptic pathways. Since those pharmacological activities were achieved after systemic administration (i.p.) of vitexin, it is plausible to affirm that this compound overcomes the blood-brain barrier, making it more attractive in terms of pharmacokinetic or even pharmaceutical perspectives. Vitexin has been found at different medicinal plants with anxiolytic and/or anti-epileptic properties (Nassiri-Asl et al., 2007; Miroddi et al., 2013). Based on this and also on our results, it is also possible to suggest that vitexin is at least partially responsible for those pharmacological activities, in most of cases and more probabilistically, acting in a synergistic way with other(s) active compound(s) present in those plants claimed as anxiolytics and/or anti-epileptics.

In the present study, anxiolytic-like effect of vitexin was confirmed by three different and complementary animal screening tests. This combined arrangement of tests allows a more reliable and complete evaluation of anxiety-related behaviors in rodents (Ramos et al., 2008). Overall results from different tests confirmed the increase of exploratory performance and the decrease of aversive behaviors of animals, indicating an anxiolytic-like effect promoted by the different doses of vitexin. Anxiolytics agents reduce protected risk assessment behaviors and increase the frequency of unprotected ethological behaviors (Benneh et al., 2018). Similar to diazepam, pretreatment with vitexin significantly decreased protected head dips and stretch attend postures and increase unprotected behavior suggesting the anxiolytic property of vitexin. In addition, it is possible to reinforce that vitexin did not caused any type of locomotor impairment or even did not produced signals of sedation on animals at same time that the anticonvulsant activity produced by vitexin is not caused by interference with locomotor capacity of the treated animals. Those conclusions are supported by the increased number of crossings at open field test and the increased number of light/dark transitions exhibited by vitexin treated animals in the light/dark box test when compared with the saline control. Locomotor impairment or severe sedation are constantly claimed as important side effects caused by different pharmacological agents used in the treatment of anxiety and epilepsy and should be avoided in the clinical management of those medical conditions (Canevini et al., 2010; Guina and Merrill, 2018). Therefore, besides the useful dual pharmacological effect (anxiolytic and anticonvulsant) vitexin presents some advantages in terms of its toxicological profile. The dual effect of vitexin at a very similar range of doses is particularly important for a potential combined and future management of both epilepsy and anxiety, which commonly appears as comorbidities and request the use of several combined drugs. However, this dual pharmacological effect is not surprising or exclusive of vitexin, since epilepsy and anxiety are partially overlapped in terms of neurochemical and neurobiological pathways and some drugs depending on the dose (like benzodiazepines) may act against both disorders despite important observed side effects.

The effect of acute intraperitoneal injection of the vitexin for the prospect of its anticonvulsant activity was evaluated using different acute seizure models in which the seizures were chemically induced. Given the possibility of false negatives results on drug screening, potential antiepileptic compounds should not be screened solely against one single model but to be tested across a few different seizure models, exploring at same time different neurotransmission pathways involved on initiation and spraying of induced seizures (Yuen and Troconiz, 2015). These type of animal models are very useful for the rapid and economical screening of new anticonvulsant drugs (Löscher, 2011). Kainic acid and NMDA have significant convulsant effects, fundamentally by activating glutamate receptors; glutamate is the most prominent excitatory neurotransmitter in the mammalian CNS (Bhandage et al., 2017). Moreover, in the current study, we explored the therapeutic potential of vitexin using the GABA_A_ receptor antagonists PTZ and picrotoxin (Colas et al., 2013). On the basis of the results presented here, acute treatment with different doses of vitexin applied against the kainic acid and NMDA seizures protocol was not effective when compared with the saline control. In contrast, vitexin exhibited a very clear dose-dependent anticonvulsant effect against seizures induced by PTZ and picrotoxin, reaching the maximum protection (100%) at higher dose of 5 mg/kg. In addition, the lower doses (1.25 and 2.5 mg/kg) delayed the onset of seizures for non-completely protected animals against seizures. The combination of these actions indicated an effective anticonvulsant effect of vitexin at least against PTZ and picrotoxin-induced seizures.

The underlying molecular mechanisms involved in the anticonvulsant or anxiolytic actions of vitexin were not the central scope of the present study. However, the selective protection offered by vitexin against GABA receptors antagonists at the expense of Glutamate receptor agonists are at least intriguing and may suggest that the modulation of GABA neurotransmission or related pathways may be involved on mode of action of vitexin. It is noteworthy to point out that different natural flavonoids and various synthetic derivatives from natural flavonoids are well known to be positive modulators of GABA_A_ receptors (Hanrahan et al., 2011; Oliveira et al., 2018). Indeed, many studies agree that vitexin may exert its CNS effects *via* GABA_A_ receptor (benzodiazepine site) modulation (Abbasi et al., 2012; Oliveira et al., 2014; World Health Organization, 2019b). However, further neurochemical investigations are required for a more complete understanding by the way vitexin exerts its pharmacological activities. The GABA neurotransmission is a common link between epilepsy and anxiety. In fact, the down regulation of GABA activity is frequently evoked on the neurobiological explanation for both disorders. Also, the up modulation of GABAergic neurotransmission is assumed on the explanation about the mode of action of some drugs such as benzodiazepines, which are well known as a GABA_A_ receptors positive allosteric modulators and commonly used for the clinical management for both disorders (Beyenburg et al., 2005).

Finally, epilepsy and anxiety frequently appear as comorbidities. Indeed, one of the main psychiatric conditions observed for epileptic patients is anxiety. Anxiety affects approximately 20% of the people diagnosed with epilepsy and is responsible for a decreased quality of life and can worsen epilepsy control (Dworetzky, 2017; World Health Organization, 2019b). The actual pharmacological arsenal used in the clinical treatment for both disorders often causes important side effects, such as cognitive/locomotor impairments and sedation, which negatively affects therapeutic adherence (Diniz et al., 2019). Thus, the prospecting/developing of new pharmacological probes is relevant especially when those new potential drugs exhibit potential advantages in terms of pharmacokinetics, toxicological, and pharmacodynamic features.

## Conclusion

The results of this study showed that vitexin possess both anxiolytic and anticonvulsant properties. Since vitexin has been found in different medicinal plants popularly claimed as anxiolytics and/or anticonvulsants, this work has both ethnopharmacological and pharmaceutical relevance. This work may be useful for the development of new pharmacological probes not only for anxiety and epilepsy patients relieve in the future but also, and indirectly, for the better understanding of pathological events underlying both anxiety and epilepsy themselves.

## Data Availability Statement

The datasets generated for this study are available on request to the corresponding author.

## Ethics Statement

The animal study was reviewed and approved by University of Ribeirão Preto Ethic Committee (11/2015).

## Author Contributions

Conceived and Designed: DO and RB. Data Collection: DO, CS and BI. Acquisition, Analysis and Interpretation of Data: DO and CS. Article Writing by: DO and RB. All authors contributed to the article and approved the submitted version.

## Funding

This work was supported by the Coordination for the Improvement of Higher Education Personnel (CAPES) and the Brazilian National Council for Scientific and Technological Development (CNPq).

## Conflict of Interest

The authors declare that the research was conducted in the absence of any commercial or financial relationships that could be construed as a potential conflict of interest.

## References

[B1] Abbasi E. Nassiri-Asl M. Shafeei M. Sheikhi M. (2012). Neuroprotective Effects of Vitexin, a Flavonoid, on Pentylenetetrazole-Induced Seizure in Rats. Chem. Biol. Drug Des. 80 (2), 274–278. 10.1111/j.1747-0285.2012.01400.x 22554436

[B2] Abbasi E. Nassiri-Asl M. Sheikhi M. Shafiee M. (2013). Effects of vitexin on scopolamine induced memory impairment in rats. Chin. J. Physiol. 56 (3), 184–189. 10.4077/CJP.2013.BAB123 23656220

[B3] Benneh C. Biney R. P. Adongo D. W. Mante P. K. Ampadu F. A. Tandoh A. (2018). Anxiolytic and Antidepressant Effects of *Maerua angolensis DC*. Stem Bark Extract in Mice. Depression Res. Treat, 1–18. 10.1155/2018/1537371 PMC6151235 30271633

[B4] Beyenburg S. Mitchell A. J. Schmidt D. Elger C. E. Reuber M. (2005). Anxiety in patients with epilepsy: Systematic review and suggestions for clinical management. Epilepsy Behav. 7 (2), 161–171. 10.1016/j.yebeh.2005.05.014 16054870

[B5] Bhandage A. K. Jin Z. Hellgren C. Korol S. V. Nowak K. Williamsson L. (2017). AMPA, NMDA and kainate glutamate receptor subunits are expressed in human peripheral blood mononuclear cells (PBMCs) where the expression of GluK4 is altered by pregnancy and GluN2D by depression in pregnant women. J. Neuroimmunol. 305, 51–58. 10.1016/j.jneuroim.2017.01.013 28284346

[B6] Bialer M. White H. S. (2010). Key factors in the discovery and development of new antiepileptic drugs. Natural Rev. Drug Discovery 9, 68–82. 10.1038/nrd2997 20043029

[B7] Can O. D. Demir Ozkay U. Ucel U. I. (2013). Anti-depressant-like effect of vitexin in BALB/c mice and evidence for the involvement of monoaminergic mechanisms. Europen J. Pharmacol. 699, 250–257. 10.1016/j.ejphar.2012.10.017 23099258

[B8] Canevini M. P. Sarro G. Galimberti C. A. Gatti G. Liccheta L. Malerba A. (2010). Relationship between adverse effects of antiepileptic drugs, number of coprescribed drugs, and drug load in a large cohort of consecutive patients with drug-refractory epilepsy. Epilepsia 51 (5), 797–804. 10.1111/j.1528-1167.2010.02520.x 20545754

[B9] Colas D. Chuluun B. Warrier D. Blank M. Wetmore D. Z. Buckmaster P. (2013). Short-term treatment with the GABAA receptor antagonist pentylenetetrazole produces a sustained pro-cognitive benefit in a mouse model of Down’s syndrome. Br. J. Pharmacol. 169 (5), 963–973. 10.1111/bph.12169 23489250 PMC3696321

[B10] Diniz T. C. Júnior R. G. O. Medeiros M. A. M. B. Silva M. G. Teles R. B. A. Menezes P. P. (2019). Anticonvulsant, sedative, anxiolytic and antidepressant activities of the essential oil of Annona vepretorum in mice: Involvement of GABAergic and serotonergic systems, (2019). Biomed. Pharmacother. 111, 1074–1087. 10.1016/j.biopha.2018.12.114 30841421

[B11] Dworetzky B. A. (2017). Worrying More about Anxiety in Patients with Epilepsy. Epilepsy Curr. 17 (6), 353–354. 10.5698/1535-7597.17.6.353 29217976 PMC5706354

[B12] England M. J. Liverman C. T. Schultz A. M. Strawbridge L. M. (2012). Epilepsy across 455 the spectrum: Promoting health and understanding. Epilepsy Behav. E&B 25 (2), 266–276. 10.1016/j.yebeh.2012.06.016 PMC3548323 23041175

[B13] Fisher P. L. Noble A. J. (2017). Anxiety and depression in people with epilepsy: The contribution of metacognitive beliefs. Seizure 50, 153–159. 10.1016/j.seizure.2017.06.012 28667910

[B14] Funchal C. Dani C. (2014). Neurosciências: Modelos Experimentais Animais. EdiPUCRS, 280.

[B15] Gandy M. Sharpe L. Perry K. N. Miller L. Thayer Z. Boseiro J. (2015). Anxiety in epilepsy: a neglected disorder. J. Psychosom. Res. 78, 149–155. 10.1016/j.jpsychores.2014.12.002 25541120

[B16] Gauthier I. Nuss P. (2015). Anxiety disorders and GABA neurotransmission: a disturbance of modulation. Neuropsychiatr. Dis. Treat. 11, 165–175. 10.2147/NDT.S58841 25653526 PMC4303399

[B17] Guimarães C. C. Oliveira D. D. Valdevite M. Fachin A. L. S. Pereira S. I. V. França S. C. (2015). The glycosylated flavonoids vitexin, isovitexin, and quercetrin isolated from Serjania erecta Radlk (Sapindaceae) leaves protect PC12 cells against amyloid-beta peptide-induced toxicity. Food Chem. Toxicol. 86, 88–94. 10.1016/j.fct.2015.09.002 26385725

[B18] Guina J. Merrill B. (2018). Benzodiazepines I: Upping the Care on Downers. J. Clin. Med. 7 (2), 1–22. 10.3390/jcm7020017 PMC5852433 29385731

[B19] Hanrahan J. R. Chebib M. Johnston G. A. R. (2011). Flavonoid modulation of GABAA receptors. Br. J. Pharmacol. 163 (2), 234–245. 10.1111/j.1476-5381.2011.01228.x 21244373 PMC3087128

[B20] Hascoet M. Bourin M. (1998). A new approach to the light/dark procedure in mice. Pharmacol. Biochem. Behav. 60, 645–653. 10.1016/s0091-3057(98)00031-8 9678648

[B21] He M. Min J. W. Kong W. L. He X. H. Li J. X. Peng B. W. (2016). A review on the pharmacological effects of vitexin and isovitexin. Fitoterapia 115, 74–85. 10.1016/j.fitote.2016.09.011 27693342

[B22] Kazdoba T. M. Hagerman R. J. Zolkowska D. Rogawski M. L. A. Crawley J. N. (2015). Evaluation of the neuroactive steroid ganaxolone on social and repetitive behaviors in the BTBR mouse model of autism. Psychopharmacology 233, 309–323. 10.1007/s00213-015-4115-7 26525567 PMC4703522

[B23] Kwon O. Park S. (2014). Depression and Anxiety in People with Epilepsy. J. Clin. Neurol. 10 (3), 175–188. 10.3988/jcn.2014.10.3.175 25045369 PMC4101093

[B24] Löscher W. (2011). Critical review of current animal models of seizures and epilepsy used in the discovery and development of new antiepileptic drugs. Seizure 20 (5), 359–368. 10.1016/j.seizure.2011.01.003 21292505

[B25] Martin E. II Ressler K. J. Binder E. Nemeroff C. B. (2009). The Neurobiology of Anxiety Disorders: Brain Imaging, Genetics, and pychoneuroendocrinology. Psychiatr. Clinics North America 32 (3), 549–575. 10.1016/j.psc.2009.05.00 PMC3684250 19716990

[B26] Masneuf S. Lowery-Gionta E. Colacicco G. Pleil K. E. Li C. Crowley N. (2014). Glutamatergic mechanisms associated with stress-induced amygdala excitability and anxiety-related behavior. Neuropharmacology 85, 190–197. 10.1016/j.neuropharm.2014.04.015 24796255 PMC4170856

[B27] Miroddi M. Calapai G. Navarra M. Minciullo P. L. Gangemi S. (2013). Passiflora incarnata L.: Ethnopharmacology, clinical application, safety and evaluation of clinical trials. J. Ethnopharmacol. 150 (3), 791–804. 10.1016/j.jep.2013.09.047 24140586

[B28] Moto F. C. O. Arsa’a A. Ngoupaye G. T. Taiwe G. S. Njapdounke J. S. K. Kandeda A. K. (2018). Anxiolytic and Antiepileptic Properties of the Aqueous Extract of *Cissus quadrangularis* (Vitaceae) in Mice Pilocarpine Model of Epilepsy. Front. Pharmacol. 9, 751. 10.3389/fphar.2018.00751 30065650 PMC6056655

[B29] Nassiri-Asl M. Shariati-Rad S. Zamansoltani F. (2007). Anticonvulsant effects of aerial parts of *Passiflora incarnata* extract in mice: involvement of benzodiazepine and opioid receptors. BMC Complement. Altern. Med. 7. 10.1186/1472-6882-7-26 PMC1973074 17686156

[B30] Oliveira D. R. Zamberlam C. R. Gaiardo R. B. Rêgo G. M. Cerutti J. M. Cavalheiro A. J. (2014). Flavones from *Erythrina falcata are* modulators of fear memory. BMC Complement. Altern. Med. 14 (1), 1–17. 10.1186/1472-6882-14-288 25096710 PMC4141959

[B31] Oliveira D. R. Todo A. H. Rêgo G. M. Cerutti J. M. Cavalheiro A. J. Rando D. G. G. (2018). Flavones-bound in Benzodiazepine Site on GABA A Receptor: Concomitant Anxiolytic-Like and Cognitive-Enhancing Effects Produced by Isovitexin and 6-C-glycoside-Diosmetin. Eur. J. Pharmacol. 15 (831), 77–86. 10.1016/j.ejphar.2018.05.004 29738701

[B32] Pellow S. Chopin P. File S. E. Briley M. (1985). Validation of open: closed arm entries in an elevated plus-maze as a measure of anxiety in the rat. J. Neurosci. Methods 14, 149–167. 10.1016/0165-0270(85)90031-7 2864480

[B33] Pinel J. P. Rovner L. II (1978). Electrode pacement and kindling: induced experimental epilepsy. Exp. Neurol. 58 (15), 35–346-202. 10.1016/0014-4886(78)90145-0 618751

[B34] Ramos A. Pereira E. Martins G. C. Wehrmeister T. D. Izidio G. S. (2008). Integrating the open field, elevated plus maze and light/dark box to assess different types of emotional behaviors in one single trial. Behav. Brain Res. 193, 277–288. 10.1016/j.bbr.2008.06.007 18590774

[B35] Salpekar J. A. Mula M. (2018). Common psychiatric comorbidities in epilepsy: How big of a problem is it? Epilepsy Behav. 18, 1–5. 10.1016/j.yebeh.2018.07.023 30149996

[B36] Walf A. A. Frye A. C. (2007). The use of the elevated plus maze as an assay of anxiety-related behavior in rodents. Nat. Protoc. 2, 322–328. 10.1038/nprot.2007.44 17406592 PMC3623971

[B37] World Health Organization (2019a). Epilepsy key facts. Available at: https://www.who.int/news-room/fact-sheets/detail/epilepsy (Accessed October 04, 2019).

[B38] World Health Organization (2019b). Epilepsy: a public health imperative. Available at: https://www.who.int/mental_health/neurology/epilepsy/report_2019/en/ (Accessed October 15, 2019).

[B39] Wu Q. Zhao C. W. Long Z. Xiao B. Feng L. (2018). Anatomy Based Networks and Topology Alteration in Seizure-Related Cognitive Outcomes. Front. Neuroanat. 12, 25. 10.3389/fnana.2018.00025 29681801 PMC5898178

[B40] Yuen E. S. M. Troconiz I. F. (2015). Can pentylenetetrazole and maximal electroshock rodent seizure models quantitatively predict antiepileptic efficacy in humans? Seizure 24, 21–27. 10.1016/j.seizure.2014.11.006 25564315

[B41] Zemdegs J. Rainer Q. Grossmann C. P. Rousseau-Ralliard D. Grynberg A. Ribeiro E. (2018). Anxiolytic- and Antidepressant-Like Effects of Fish Oil-Enriched Diet in Brain-Derived Neurotrophic Factor Deficient Mice. Front. Neurosci. 12:2018.00974. 10.3389/fnins.2018.00974 PMC6308198 30622454

